# The Preliminary Effects of a Multi-Recess School Intervention: Using Accelerometers to Measure Physical Activity Patterns in Elementary Children

**DOI:** 10.3390/ijerph17238919

**Published:** 2020-11-30

**Authors:** David Farbo, Laura C. Maler, Deborah J. Rhea

**Affiliations:** Kinesiology Department, Texas Christian University, Fort Worth, TX 76129, USA; l.clark@tcu.edu

**Keywords:** accelerometers, children, intervention, physical activity, recess, school, steps

## Abstract

This pilot study used accelerometers to investigate the effectiveness of a multiple recess school intervention on physical activity patterns in younger elementary children using a post-test only with nonequivalent groups design. First and second grade students (*N* = 157) participating in a larger study, the LiiNK Project^®^ (Let’s inspire innovation ‘N Kids), wore accelerometers for the duration of the school day for two weeks to measure physical activity intensity and number of steps taken daily. Students attended either an intervention school (*N* = 90), participating in four 15-min unstructured, outdoor recesses and one 15-min character development lesson daily, or a control school (*N* = 67), participating in two 15-min unstructured, outdoor recesses daily and no character development program. The intervention students, grades 1 and 2, took more steps (*p* < 0.001) and time spent in moderate (*p* < 0.001) and vigorous (*p* < 0.001) physical activity (MVPA) than the control school students. Intervention students averaged approximately 900 more steps per day than the control school students. These results show young children given 60 min of recess daily continue to increase physical activity patterns over those with 30 min of recess daily. Next steps are to evaluate if children demonstrate healthier body fat levels as a result of these higher patterns of MVPA daily.

## 1. Introduction

The prevalence of childhood obesity has more than doubled over the past 40 years and is now considered a “public health crisis” in the United States [1]. This continual rise in childhood obesity is alarming because of the health risks associated with obesity, such as hypertension, diabetes, hyperlipidemia, asthma, and cardiovascular disease as a child and as an adult [2], as well as a lowered ability to fight illnesses once contracting them [3]. Sedentary lifestyles and stress associated with the continuous technology demand known as technostress have been identified as two leading reasons for approximately 13.7 million children between the ages of 6–19 being overweight or obese in the United States today [4,5]. As we experience different types of diseases and health concerns, it is necessary to further evaluate how sedentary lifestyles and technostress influence the health of the nation and methods to alter obesity trends.

Sedentary lifestyles have become the new normal in today’s society, leading researchers to concentrate more on strategies that reverse sedentary trends among adults to curb obesity. However, the root of the obesity problem has begun to impact younger populations over the years, which in turn creates more of a health risk for our workforce and for longevity as we age. Research shows inactive and overweight children will become unhealthy adults with limited abilities to lead productive lives since they encounter many physical and mental ailments as a result of obesity [6]. In addition, this generation of children may be the first generation to die before their parents because of the chronic diseases associated with sedentary lifestyles [7]. Additionally, increases in technology are a significant contributor to sedentary lifestyles as children between the ages of 8 and 18 can spend up to 7 h per day with various media devices [8]. This is concerning as high amounts of screen time increase the risk of obesity in children [9]. Limiting sedentary behaviors and increasing physical activity (PA) have been identified as primary childhood obesity prevention methods [10].

The CDC recommends that children achieve at least 60-min of moderate to vigorous physical activity (MVPA) daily in order to maintain healthy lifestyles and decrease the chances of becoming obese [4]. When children are able to meet the recommended 60 min of MVPA per day, it will equate to approximately 13,000 steps in males and 11,000 steps in females [11]. However, only 22% of children between the ages of 6–19 reach these guidelines as sedentary lifestyles have become more normalized and accepted [4,12]. Female children and adolescents face a greater obesity risk as they consistently engage in lower levels of physical activity (PA) than their male counterparts, with some averaging 10–20% lower MVPA each day [13,14]. The school setting of 40 years ago offered an active lifestyle solution for children by providing play for at least 60-min daily while promoting a rigorous classroom climate [15]. Today, educators have trended away from outdoor play opportunities and moved into a much more sedentary learning climate, the classroom, for the majority of the day [16,17]. This learning climate mindset has produced many negative consequences associated with limited recess and PA in schools for children.

When schools do offer recess, it is usually offered for less than 20 min daily and is more structured, where children are introduced to games and activities and given specific equipment to use. This prevents children the opportunities to be creative and problem solve independently [18]. Children are also more heavily influenced by the adult’s interpretation of what play should be [19]. Conversely, unstructured, outdoor recess is defined as self-directed and self-controlled without predetermined rules or influence from adults in a safe environment [15,20]. In this environment, children have the flexibility to engage in different types of play that provide them with opportunities to explore, be creative, challenge themselves, develop body control, re-energize their brains, socialize with their peers, and decrease anxiety levels [15,16,20].

School-based PA interventions have been implemented to promote PA, including recess and classroom interventions [21]. Many of the PA or recess interventions can be implemented on a larger scale, but sadly, most focus on 6 weeks or less to gather data or outcomes and are less frequent during school hours and more frequent in after school programs [22]. When a researcher studies a classroom-based PA intervention, it typically means that an instructional break is introduced in the classroom for 3–5 min to engage students in a short movement activity like “go noodle” [21,23]. Interventions outside of the classroom usually involve adding recess during the school day to the lunch period or before school. Studies in these settings report a 2%–12% increase in MVPA during the school day and acute improvements in attention, classroom behavior, and academic achievement [23,24]. However, the results of both types of interventions are inconsistent due to variability in the amount of time students have to play or be active and the length of the intervention [24]. In addition, these interventions often implement structured PA activities that fail to include important aspects of social and emotional development.

Outdoor, unstructured play provides a different focus on the whole child than indoor or structured recesses do [15]. The LiiNK (Let’s inspire innovation N’Kids) Project, a whole child school intervention, focuses on four 15-min outdoor, unstructured play breaks in schools daily as well as a 15-min daily character lesson that emphasizes empathy over bullying, respect, honesty, and trust elements that transfer to the playground and the classroom. The LiiNK intervention has been able to counter other acute studies that promote structured, short-term activity [24] by producing longitudinal improvement in many aspects of the whole child, including healthy weight, good classroom behavior, character development, positive emotions, academic achievement, and attentional focus across diverse public and private school settings [25,26]. Additionally, PA patterns of private school children involved with this intervention were examined in a preliminary study using pedometers. The study showed the intervention children took significantly more steps during the school day than control school students [27]. However, using a more efficient measurement device, such as an accelerometer, would provide a more accurate way of measuring steps and allow for comparison of activity intensity levels. Other studies have investigated MVPA of children using accelerometers during the school day, but no other interventions have implemented four 15-min outdoor, unstructured play breaks daily for a full school year or more.

Therefore, the first research question asked if there were differences in steps taken and time spent in MVPA per day between two younger elementary school groups. It was hypothesized that the intervention school students would demonstrate significantly higher steps and time spent in MVPA than the control school students during the school day. The second research question asked if there were gender and grade level differences by group for steps taken and time spent in MVPA per day. The second hypothesis was that there would be grade level differences in steps and MVPA per day between the two groups. A third hypothesis was there would be gender differences in steps and MVPA per day between the two groups.

## 2. Materials and Methods

### 2.1. Participants

This preliminary study used a post-test only with nonequivalent groups design. Participants were selected from a convenience sample of two North Texas public elementary schools participating in a longitudinal intervention called The LiiNK Project. This intervention was approved through a partnership between the university research team and the school district to measure whole child initiatives. If a child was in one of the two schools chosen for accelerometer data collection, the child could decline participation in this study, but would still have to participate in the two recesses daily in a control school or four recesses daily in the intervention school due to the nature of the Memorandum of Understanding intervention agreement.

The school superintendent preassigned which school would receive the treatment and which would be the control prior to intervention implementation. All students in each grade level of the preassigned schools encompassed the intervention or the control group. This intervention has been implemented over the past three years in different schools and always begins in kindergarten and first grade, then advances to second grade in the next year and so on. This study’s participants were from year 2 of the intervention. All students were asked to participate if they were in first or second grade at the intervention or control schools and followed their normal classroom schedule. The only exclusion criteria were if a student were injured or had a physical ailment that would hinder their physical activity during recess.

A priori power analysis was conducted using G*Power 3.1.9.2 to determine correct sample size based on main and interaction effects between group, school, and gender using a medium effect size (f^2^ = 0.25) and an alpha of 0.05. The results of the G*Power analysis revealed that a total sample of 153 participants with two equal sized groups of *n* = 77 was required to achieve a power of 0.95 [28]. Originally, 200 first and second grade students, with parent/legal guardian consent, volunteered to participate in this study to meet the G*Power requirements and account for any sample attrition rates. Eleven students (1 intervention; 10 control) withdrew from the study, while 24 students (1 intervention; 23 control) did not meet the minimum wear time of four 7-h school days. Most of the children who withdrew from the study did so because they did not like wearing the accelerometer on their wrist. Finally, eight students in the intervention group were removed from the final analysis due to outliers. Therefore, 157 students (Intervention = 90, Control = 67) of the original 200 students completed the study which resulted in a 21.5% attrition rate for the sample.

The intervention school (*N* = 90) was comprised of 46 first graders (23 male; 23 female) and 44 second graders (20 male; 24 female). The control school (*N* = 67) was comprised of 35 first graders (16 male; 19 female) and 32 second graders (16 male; 16 female). The intervention school students received four 15-min recess periods and a 15-min character development lesson daily. The control school children received two 15-min recesses daily and no character development lessons daily or weekly. Both schools came from the same district located in a suburb of North Texas, were in close proximity to each other, and their students consisted of 40% white, 40% Hispanic, 15% black, and 5% other.

### 2.2. Measure

#### Sedentary Time and Physical Activity Assessment

The Actigraph wGT3X-BT (ActiGraph, Pensacola, FL, USA) accelerometer was used to measure total steps and time spent in different PA intensities including sedentary, light, moderate, and vigorous PA. The wGT3X-BT is designed to be worn on the participant’s non-dominant wrist to accurately track movement [29] and has shown to be reliable and valid with children between the ages of 5 and 8 years [30]. This type of accelerometer uses tri-axial acceleration sensors (vertical, horizontal, & perpendicular) to detect changes in the device’s orientation during different time sampling intervals, known as epochs. The device is then able to calculate steps and time spent in different intensities of PA by the number of changes in orientation the device was able to detect during each time sampling interval.

Steps. Steps are tracked by the accelerometer recording the number of changes in vertical or horizontal orientation during each time sampling interval. An algorithm present in the device then uses the raw data recorded by changes in position and converts that into total steps for the collection window. This data can then be categorized into different time segments of the day to determine the number of steps that were taken during school hours.

Time and Intensity. Time spent in different PA intensities is calculated by the device recording changes in orientation and converting those to counts per minute (CPM). The device then uses metabolic equivalents, age, height, and weight to determine CPM for the time collection period. Once CPM is calculated, a filter is used to categorize sedentary, light, moderate, and vigorous activity. For the purpose of this study, the Puyau filter was used. Sedentary activity was set at 0–799 CPM, light was 800–3199, moderate was 3200–8199, and vigorous was anything above 8200 CPM [31].

The average amount of time spent per school day in sedentary, light, moderate, and vigorous activity, as well as the number of steps taken per school day, were used in the final analysis. Other research using accelerometers reports that there is a wide range of minimum wear time needed to reflect regular PA in children [32]. The most commonly used accelerometer wear time ranges in children from three to four days of 6–10 h of data [32]. Students in the current study had to wear the device for a minimum of four, 7-h school days for their data to be valid. The Actigraph accelerometer program has a “wear time validation” feature that will tell the researcher exactly how long students wore the device during the collection period. This feature was used to determine if participants had at least four days of data to be used in the final analysis. The program also allows researchers to set a time frame to reflect the school day so that an accurate assessment of PA intensity and steps can be tracked and downloaded by the device. Each collection day time-frame was set for the beginning of the school day to the end of the school day which equated to seven hours per school per day.

### 2.3. Procedures

#### LiiNK Intervention

Let’s Inspire Innovation ‘N Kids (The LiiNK^®^ Project) is a university researcher directed intervention modeled after the Finnish education system [20] who learned it from U.S. school practices 30–40 years ago [33]. The LiiNK Project’s primary focus is to improve the whole child by strategically increasing the number of recesses daily and teaching a character lesson daily [20]. The recess component of this intervention requires the school to engage in two 15-min recess breaks typically before lunch and two 15-min recess breaks after lunch. The LiiNK recess intervention is defined as outdoors, unstructured, and children are able to engage in free play with no influence from adults. At no time are children required to be physically active during these recesses daily. In addition, recess cannot be withheld from students due to disciplinary actions or a need for tutoring. Therefore, number of recess minutes dedicated to physical activity could still look very different based on a child’s need to move or motivation to move. The second component requires teachers to deliver daily 15-min character lessons from a curriculum called Positive Action (PosA) to enhance social and emotional skills. The PosA curriculum counters the propensity for bullying on the playground and in the school by focusing on positive thoughts, actions, and feelings throughout the school day with stories introduced daily. The six character traits emphasized are empathy, trust, honesty, confidence, self-esteem, and respect [34]. The third component, which is required in the spring prior to launching the intervention in the fall, is for teachers and administrators to participate in three full day trainings in order to learn: (1) the research behind the importance of the outdoors and unstructured play; (2) the difference between quantity and quality teaching methods; (3) how to transition children to recess and back quickly, and (4) how to teach the character curriculum [20].

This study’s intervention school and control school were from the same district and had similar demographics, i.e., race, school size, number of classrooms, socio-economic status (SES), and title I status. This district’s non-intervention schools were all having 30 min of recess daily as part of a school district policy. Therefore, the control school for this study had one recess every morning for 15 min and one recess every afternoon for 15 min. Even though the control school had at least 10 min more recess time daily than the national recommended average [35], it was still half of the amount of recess that the intervention school was receiving daily.

### 2.4. Accelerometer Intervention

The LiiNK intervention was approved by the University Institutional Review Board (IRB-1411–113-1702AM) and all ethical principles of the Declaration of Helsinki were met. Informed consent packets were signed to approve or decline participation by all parents/legal guardians and returned before the start of the accelerometer study. Teachers and parents/guardians who approved of their children’s participation attended an information meeting led by the lead researcher before data collection to review the objectives, procedures, and routines required for the child. Child assent was also obtained. If at any time any child chose to end participation in the study, the child was allowed to do so, but would still continue to receive two or four recesses daily. Each participant wore an accelerometer to measure PA patterns during the school day, approximately seven hours of data daily. First graders wore the devices for two weeks, followed by the second graders wearing them for two weeks. Each student was assigned an accelerometer number so that the data could be tracked for that specific student throughout the study.

Researchers were present simultaneously on the first data collection day at the intervention and control schools to hand out devices, help secure the device to the student’s wrist, provide group instructions, and answer any procedural questions teachers and students might have. The participants were instructed to wear the devices on their non-dominant wrist from the time school started that first scheduled Monday morning until the end of the day on Friday of the same week. The researchers did not have the children take the accelerometers off daily; they wore the bands continually from Monday through Friday. When the devices were collected each Friday, the lead researcher uploaded the data to a computer to ensure that each device was functioning properly, then data was backed up to a secure computer drive, and each device was charged to be used again for the next collection week. If a student had a device that was not functioning properly, a new device was given to the student to be used for the second data collection week. The same procedures were utilized to collect the child’s data during the second week of collection (five total days). At the conclusion of the second week, the same procedures were used to collect the devices and download the children’s data. The devices were then reprogrammed, given new student ID numbers, and re-distributed to the next grade level for their two weeks of data collection. At the conclusion of the collection period, the lead researcher combined the data from both grade levels into one master data file for final analysis.

Class schedules and weather conditions were collected to note any abnormal activities that occurred during the school day that would interfere with recess or cause an abnormal change in PA. No abnormal weather days affected the schools or recesses offered during collection periods. If any data collection days were lost due to field trips, cancellations, or assemblies, that day was made up between one and three weeks after the initial scheduled week. Students had to wear the device for 420 min (7 h) daily for that day’s data to be used in the final analysis. Due to complications with many of the devices, the researchers were only able to analyze four of the scheduled 10 days of data since that was the number of days consistent across all students in the study.

### 2.5. Data Analysis

Data was cleaned and coded in Microsoft Excel^®^ and then analyzed using IBM SPSS^®^ (Armonk, NY, USA) statistics software version 16.0. The average number of minutes in different PA intensities and steps per day were calculated from the total PA levels during the two-week collection period for each group. The first four days of data that met the minimum wear time requirements in the two week collection period were used to average the per day data for each student and the remaining days were excluded. Due to unequal groups following the power analysis results, proper statistical analyses were executed to account for the unequal groups. Descriptive statistics were used to determine the means and standard deviations of the demographic data (gender, grade, and total sample). The first, second, and third hypotheses were tested using a multivariate analysis of variance (MANOVA) to determine differences and interactions between group, gender, and grade with a significance value set at *p* < 0.05. Follow up one-way MANOVA and ANOVA were then used to determine differences for any interaction or main effects from the MANOVA, also with a significance value set at *p* < 0.05.

## 3. Results

Descriptive statistics of steps and PA intensity per day were conducted to describe the sample as a whole and review the trends of the data before separating participants by groups. Second grade students displayed higher steps and minutes in MVPA, and first grade students showed higher minutes in sedentary and light PA. Males and females displayed similar patterns of PA intensity per day, but males consistently took more steps than females. Means and standard deviations (SD) of the sample by grade, gender, and the total group are provided in Table 1.

### 3.1. Research Question 1 Analysis

A one-way MANOVA was performed to determine if there were group differences with steps taken and time spent in MVPA during the school day. All assumptions of MANOVA were met including normality, homogeneity, linearity, and no outliers. The MANOVA revealed the intervention and control groups were different in this study, Wilks Lambda = 0.685, F(4152) = 17.50, *p* < 0.001.

#### 3.1.1. Steps

**Figure 1:**
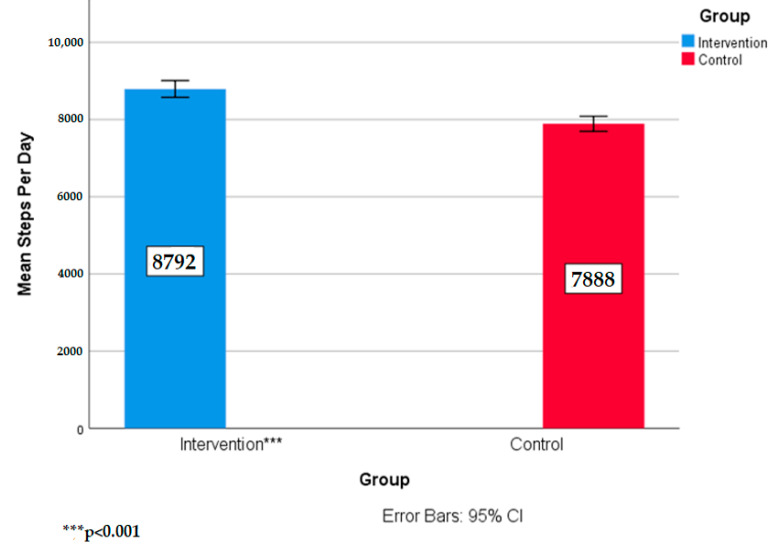
Means and standard deviations of steps per day by group.

Follow up analyses revealed that group had a significant effect on steps per day, F(1155) = 34.97, *p* < 0.001, n^2^ = 0.18. Intervention students took significantly more steps during the day than control school students. These results support the first hypothesis that intervention students would demonstrate significantly higher steps than control school students. Intervention students were averaging approximately 900 more steps per day than the control school students. Figure 1 shows the means and standard deviations of steps per day per group.

#### 3.1.2. MVPA

**Figure 2:**
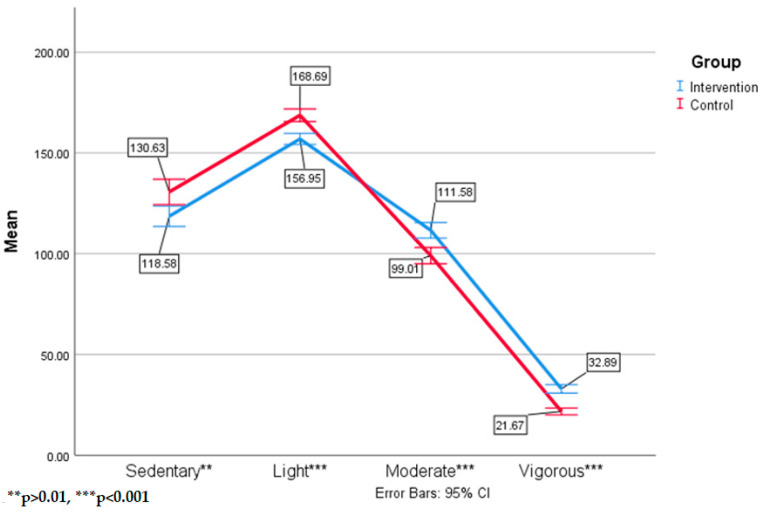
Means and standard deviations of time by physical activity (PA) intensities by group.

Follow up analyses for PA intensity differences further supported the first hypothesis, finding intervention students spent significantly more time in moderate, F(1155) = 19.42, *p* = <0.001, n^2^ = 0.11 and vigorous, F(1155) = 61.49, *p* < 0.001, n^2^ = 0.28, PA throughout the school day than the control group students. Control group students spent significantly more time in sedentary, F(1155) = 9.03, *p* = 0.003, n^2^ = 0.10, and light, F(1155) = 30.93, *p* < 0.001, n^2^ = 0.17, activities. Figure 2 shows the means and standard deviations of PA intensity minutes per day by group.

### 3.2. Research Question 2 Analysis

A 2 × 2 × 2 MANOVA was performed to answer the second research question on the dependent variables of minutes in each intensity (sedentary, light, moderate, vigorous) category and steps per day; while group, grade, and gender were identified as the independent variables. The MANOVA revealed a significant interaction effect for group by grade, Wilks Lambda = 0.896, F(4146) = 4.25, *p* = 0.003 and main effects for grade, Wilks Lambda = 0.859, F(4146) = 6.00, *p* < 0.001, and gender, Wilks Lambda = 0.829, F(4146) = 7.53, *p* < 0.001.

#### 3.2.1. Steps

**Figure 3:**
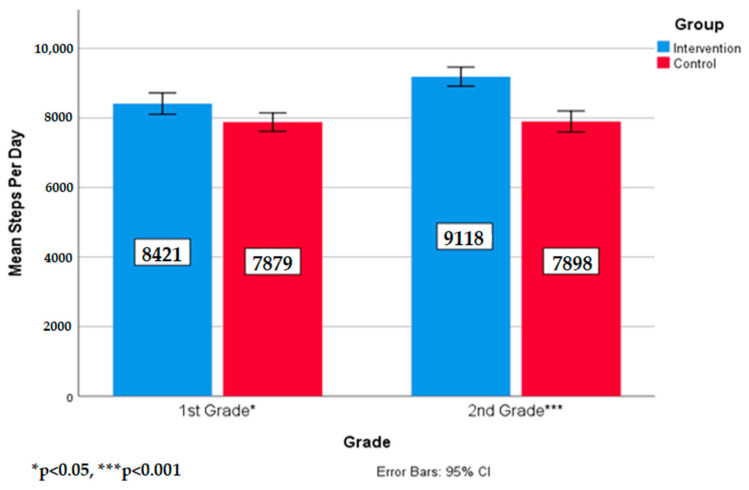
Means and standard deviations of steps per day by group by grade.

For the group by grade interaction, separate one-way MANOVAs were run to determine between group differences within each grade level. The intervention school first graders, F(1, 79) = 2.45, *p* = 0.01, n^2^ = 0.10, and second graders, F(1,74) = 40.24, *p* < 0.001, n^2^ = 0.35, took significantly more steps than control school students in the same grade levels. These results support the second hypothesis that there would be grade level differences in steps per day between the two groups. Figure 3 shows the means and standard deviations of steps per day for the school by grade level interaction.

**Figure 4:**
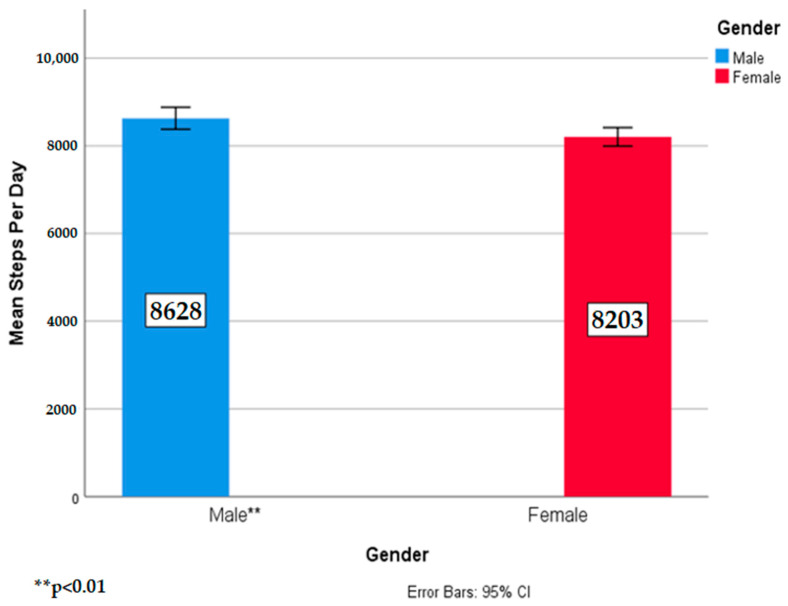
Means and standard deviations of steps per day by gender.

Follow up analysis for the main effect of gender revealed that males (*p* = 0.002) took significantly more steps than females. These results refute the third hypothesis that intervention males and females would take more steps than the control school males and females since there was no school by gender interaction effect. However, these results show that males did take more steps than females when offered either 30 or 60 min of outdoor, unstructured play during the school day. Figure 4 shows the main effect of gender.

#### 3.2.2. MVPA

**Figure 5:**
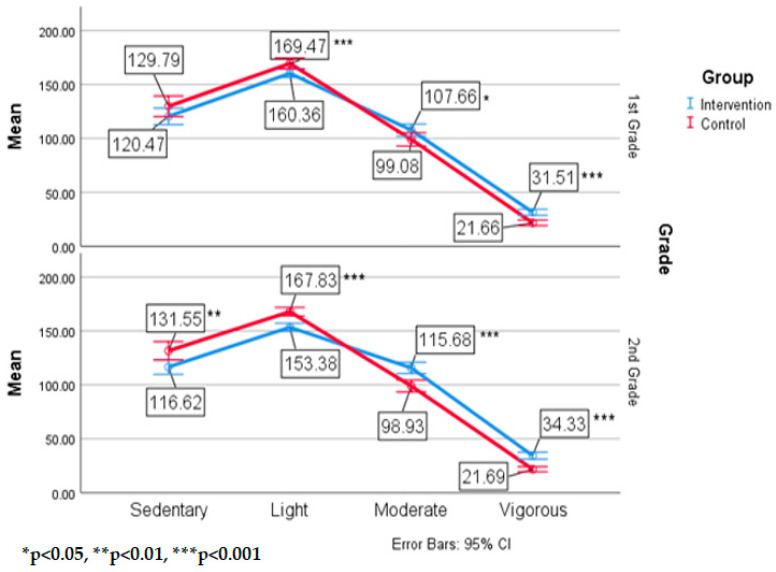
Means and standard deviations of MVPA by group by grade.

For first grade students, the follow up analysis revealed that intervention students spent significantly more minutes in moderate, F(1,79) = 4.28, *p* = 0.04, n^2^ = 0.10, and vigorous, F(1,79) = 26.19, *p* < 0.001, n^2^ = 0.25, PA than control school students. Control school first graders spent significantly more time in light PA, F(1,79) = 8.63, *p* = 0.004, n^2^ = 0.10 than the intervention school first graders. In second grade, intervention students also had significantly more time in moderate, F(1,74) = 19.25, *p* < 0.001, n^2^ = 0.21, and vigorous PA, F(1,74) = 35.45, *p* < 0.001, n^2^ = 0.30, while control school students had significantly more time spent in sedentary, F(1,74) = 7.74, *p* = 0.007, n^2^ = 0.10, and light PA F(1,74) = 27.84, *p* < 0.001, n^2^ = 0.27. These results further support the second hypothesis which stated that there would be grade level differences in MVPA between the two groups. Figure 5 shows the means and standard deviations of the school by grade interaction.

**Figure 6:**
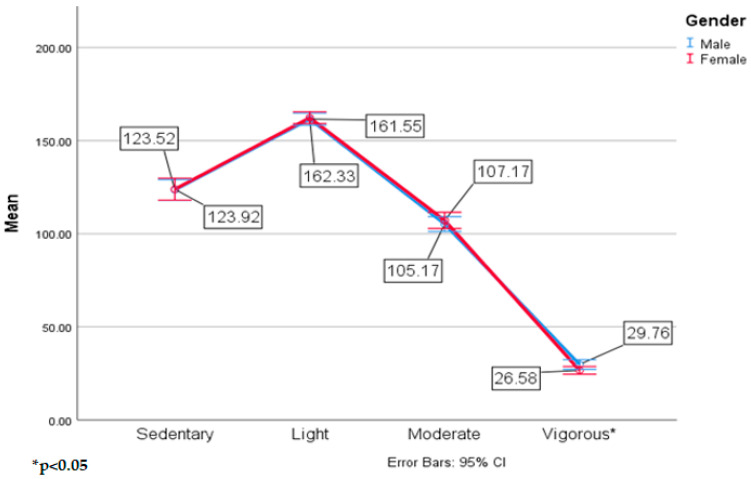
Means and standard deviations of MVPA by gender.

Follow up analysis for gender revealed that males spent significantly more time in vigorous PA (*p* = 0.03) than female students. There were no significant differences by gender in sedentary, light, and moderate activity. The third hypothesis stated that males and females in the intervention school would demonstrate higher MVPA than all students at the control school. These results refute the third hypothesis since there was no group by gender interaction effect in the MANOVA. However, these results show that males will demonstrate greater time in vigorous activity and females will demonstrate more time in moderate PA when given 30 or 60 min of outdoor, unstructured, play. The average amount of time spent in MVPA when comparing males and females was actually similar (~133 min). Figure 6 shows the means and standard deviations of the main effect of gender.

## 4. Discussion

This preliminary study, using accelerometers, sought to examine PA patterns between two schools who have 30 min or 60 min of recess daily. Educators and parents around the country are pushing for at least 20–30 min of recess daily in schools. However, it is unclear from previous research if 30 min of recess is sufficient to meet movement recommendations provided by the CDC [36,37]. With the growing number of children engaged in home sedentary activity due to limited PA opportunities, it has become more important that children try to reach their movement goals during school. This was the first study of its kind to examine 60 min of recess compared to 30 min of recess for step counts and MVPA in children. Since this intervention requires unstructured play opportunities, children are not pushed to be physically active. Therefore, the accelerometer data collected in this initial study is important to understand whether children will be active if not coerced to do so.

The purpose of this pilot study was to compare the children’s daily step counts and MVPA differences between children who received a recess intervention of four unstructured, outdoor play breaks daily and those who received two recess breaks daily. Intervention students demonstrated significantly higher step counts and time in MVPA than the control school students throughout the school day. This preliminary study, along with other studies have shown that when children engage in 30 min or more of play daily, step counts will increase [36,37]. Additionally, when accelerometers are used to measure step counts, children will take approximately 4000–6000 steps during school when only receiving one 20 min recess period per day [37]. This study showed that students with 60 min of unstructured outdoor play breaks will take ~8700 steps per day, which is significantly more than that seen in a normal elementary school day [37]. When schools implement PA interventions, whether it be inside or outside of the classroom, they typically only report a 500–1000 per day step increase in their students [36,38]. Our results show a similar difference when comparing the two groups, as intervention students took 900 more steps per day than the control students. However, considering that the control school does not represent a typical elementary school schedule and no baseline data was collected, the differences in this study may be more substantial than what other PA interventions are showing in their results. If the baseline data in these schools reflected what is typically seen in elementary schools with only one recess, implementing four recesses during the school day may increase step counts by ~3000 steps [37].

The results also revealed that the intervention students in first and second grades took significantly more steps than the control school students in the same grades. First grade and second grade students that follow a normal school schedule with only one recess are shown to take ~5200 steps per day [36]. First grade students in the intervention school averaged ~8400 steps per day while second grade intervention students took ~9100 steps per day. Our findings show that 60 min of outdoor unstructured play may result in a ~60% increase in steps when compared to only one recess period per day [36]. Interestingly, the difference between grade levels in the intervention school was ~700 steps, while the control school did not show any difference in steps between grade levels. This could mean that intervention students are becoming more active as they age while the control students may have already hit a threshold of steps per day by second grade.

This study did not show gender differences by group, but these results did show males took significantly more steps than females during the school day across both groups. Previous research has shown that males are often more active than females and will take ~1000 more steps per day during school [36,37]. The findings in the current study show a similar pattern as males took ~600 more steps per day than females, regardless if they were offered 30 or 60 min of outdoor, unstructured play. However, the difference between genders was much smaller than that seen in other studies, suggesting that females can display similar PA patterns to males if they are given more opportunities to be active.

One might argue that the increase of steps could be a result of more transitions to and from the playground in the intervention school because of the extra recesses. If steps alone were the indicator, we might think there is not enough difference between the two groups to worry about adding more recess to the day. However, the PA intensity data provides context to the differences in steps and is able to refute the idea that students were experiencing more activity from the transitions alone. Intervention school transitions to recess are orderly, and children are able to go from the classroom to the playground in 90 s or less without any running or inappropriate behavior in the hallways. The intervention school students achieved ~25 min more MVPA and ~25 min less sedentary and light PA per day than control school students and this large difference could not be a result of the extra transitions to recess. In a normal school schedule with one recess during the school day, children will average ~20 min of MVPA per day [39]. Studies that implement PA interventions in schools report anywhere from a 2%–12% increase in MVPA [23,24]. Students in the intervention schools are achieving significantly more MVPA during the school day (~145 min) compared to other studies that implement PA interventions during school (~30 min) [21,40]. This finding suggests that outdoor, unstructured free play is more effective to increase steps and MVPA in children than structured classroom-based PA (e.g., Go Noodle) or physical education interventions [21,40]. In addition, the intervention school students had an 18% increase in MVPA and a 10% decrease in sedentary and light PA over the control schools students. Previous studies reported that students can spend between 300–350 min in either sedentary or light PA when only provided one 20 min recess in a seven hour school day [41]. The control school children in this study reported similar results to previous studies of an average of 300 min per day in sedentary and light PA, which was significantly more than the intervention school students reporting 275 min daily. The difference in MVPA could be higher if the control school students in the current study had only implemented 0–20 min of outdoor, unstructured play daily which is seen in most U.S. elementary schools as opposed to the 30 min they received [16,20].

When examining grade level differences, first graders who engaged in 60 min of recess daily, recorded a 15% MVPA increase and 7% decrease in sedentary and light PA over those students engaged in 30 min of recess daily. Intervention second graders recorded a 20% MVPA increase and 10% sedentary and light PA decrease over those students in the control school. Previous studies show that students are likely to decrease their daily PA levels after age 5 and this trend will continue until they reach adolescence and then their PA patterns seem to plateau [42]. A reason for the decrease during these ages may be due in part to students attending schools and sitting for longer periods of time during the day. The control students in the current study support these previous findings as they did not demonstrate any differences in MVPA between first and second grade students and spent similar times in sedentary and light PA. However, the intervention school second graders demonstrated ~10 min more MVPA than first graders daily which does not support earlier research [42]. Adding recess to the school day may be the key to maintaining PA patterns of children as they age and preventing sedentary lifestyles to be adopted.

Vigorous PA is the only category in which males displayed significantly higher numbers than females. Previous research has shown that males will average between 5–10 min more MVPA during the school day compared to girls [39]. Both males and females spent a similar amount of time being active with ~130 min of MVPA during the school day, which contradicts previous research that females will typically be more inactive than males [13]. The female students in this study showed that they will achieve the same amount of MVPA as boys if they are given more opportunities to be active during the school day. This finding is important when trying to increase MVPA in females to prevent an increase in overweight or obese percentages.

Although there was not a large percentage step and MVPA gap between the two groups, the research states that 60 min of physical activity is needed daily to enhance a child’s health. Only providing students with 30 min of recess daily automatically presents a barrier from them meeting this goal during school. High levels of steps and MVPA among children decreases their chances of becoming obese and developing hypertension, diabetes, asthma, cardiovascular disease, and other health related diseases [43]. In addition to the physical benefits of increasing time for outdoor, unstructured play, there are many mental health and developmental benefits for children. Short bouts of PA are shown to increase cognition, attention, and academic performance in elementary aged students [24,44]. Play in an outdoor setting is shown to improve physical, social, emotional, and behavioral development in children and is an essential piece to whole child development [15]. This pilot study showed there is indeed an advantage of 60 min of recess daily over 30 min of recess daily. Since children are in school nine months each year, these differences paint a much different picture when examining long term effects of the intervention on PA and potential obesity rates. These results open the door for future researchers to examine the true physical and psychological health benefits that either 60 min or 30 min of recess could provide to students. These results are especially important for schools and teachers that shorten or even eliminate recess from their curriculum. Regardless if they were given 30 or 60 min, children used their recess time to move and burn off extra energy that was built up in the classroom. Children with increased time for outdoor, unstructured free play may experience less negative health effects and greater whole child development than students who are not given time for play [16,20]. Rather than improving academic performance in the classroom, taking recess away from children can have a significant effect on their physical, social, emotional, and behavioral development as they age. Based on these results, schools across the country should try to add additional time for recess during the school day for their students.

Previous research on step counts and MVPA in children shows that they will reach approximately 11,000–13,000 steps when they are able to achieve 60 min of MVPA [11]. The data in the current study contradicts these findings as students in the intervention were achieving ~140 min of MVPA per day, but only taking 9000 steps. This finding has implications. First, step counts alone are not a true indicator of MVPA in children. As opposed to adults, children have a higher threshold that they need to meet in order to reach the moderate to vigorous PA levels. Providing children with more opportunities for outdoor, unstructured play allows children to reach that moderate to vigorous level more often during the day. Second, this data shows the sedentary nature of elementary schools across the United States. Without any opportunities for outdoor, unstructured play, children may struggle to even reach 5000 steps while they are in school. This data shows that children are using their recess time to move as much as possible after being sedentary in the classroom. Lastly, the study showed some benefits for 30 min of recess daily, but there are still significantly more benefits for 60 min of recess compared to 30 min of recess daily.

### 4.1. Limitations

There are some limitations of the study that need to be addressed. The biggest limitation is that this was a preliminary study examining whether 60 min of recess daily would be more beneficial than 30 min. Preliminary evidence highlights 60 min of recess in schools does record more steps and MVPA than 30 min of recess. However, due to the low number of children participating in this data collection, further research is needed. There are also many other variables, such as body mass index (BMI; height/weight ratio score) and playground design, that were not measured in the current study which could limit the results. For example, students with a higher BMI typically report less movement than normal weight students. Because a pre-test was not collected, the post test results could not rule out whether the control group was more overweight or obese when the intervention year started, therefore it is uncertain whether movement patterns were a true indicator of the step and MVPA differences between the two groups.

The number of days that students wore the accelerometers is a limitation of this study. It was first proposed that the students would wear the device for ten school days. However, due to complications with the device, only four days of data were analyzed per student. This short time span limits the results of this study as those four days may not reflect the regular PA habits of children for longer periods of time. Another limitation is that the control group experienced two 15 min recess breaks during the day, which does not represent a true control for this population. This is because the school district under review understood the benefits of unstructured, outdoor play and chose to implement the LiiNK Project across the district in upcoming years. As a result, administrators wanted to offer the control schools some form of increased outdoor, unstructured play so that all children in the district could benefit. Most schools in America have, on average, one recess of approximately 15–20 min daily, so the differences between groups may be more significant if this control school had been more reflective of other elementary schools across the country.

Another limitation was the inability to capture PA patterns at recess only. Capturing PA data during recess only would undoubtedly prove that 60 min of outdoor, unstructured play would result in more steps and MVPA in children than 30 min. Unfortunately, this was not a possibility unless researchers were present in schools for every recess period throughout the day. The devices are capable of isolating different times of the day to capture PA data and could be set using the class schedule provided by teachers. However, if teachers were off the schedule by even a minute, the device would capture activity that was not taking part at recess and could therefore skew the results. Because of this, all students in the intervention and control schools had the exact same start and end time of data collection for each day to ensure there was consistency throughout. In addition, the cut off points provided by the Puyau filter used to categorize CPM into MVPA is a limitation of these findings. The thresholds of this filter may have been too lenient and the different intensities of PA may have been categorized incorrectly. For example, if the threshold for moderate PA was set too low, PA that was really in the light category may have been categorized as moderate PA. The results of the current study may not have the ability to be compared with similar studies if a different filter was used.

Finally, the small effect sizes observed in the analyses are another limitation of the current study. This was most likely due to having a larger sample size and somewhat marginal differences when examining the data by day. For example, although significant, the difference between vigorous PA in males and females is only ~3 min. However, these results become much more substantial when examining the longitudinal effects of the LiiNK Project since these students will experience these increases in PA on a daily, weekly, and yearly basis. The marginal differences seen in some instances for the current study would be much larger if the collection days were combined over a longer period of time.

### 4.2. Future Directions

Future studies should increase the number of days the devices are worn. At the beginning of this study, the aim was to collect 10 days of data for each participant. However, due to attrition and device malfunction, only four days of data were collected. Examining a longer period of days could present a better representation of the PA patterns of children. In addition, this study did not track the PA patterns of children when they were at home. Future studies should include at home PA data to determine if having more opportunities to be active and outdoors during the school day actually translates to more active time outdoors and less technology/sedentary time at home. Finally, the intervention effects of increased MVPA on body composition, strength, agility, and motor skills are unknown. Future research should explore the impact of multiple recess and MVPA data on improvements of those measures.

## 5. Conclusions

The results of this study exhibit that 60 min of outdoor recess will produce higher step counts and minutes spent in MVPA during the school day than 30 min of recess. In addition, providing students with additional outdoor play opportunities during school may prevent them from adopting sedentary behaviors as they age. Finally, gender differences in PA may be eliminated if females are given more opportunities for outdoor, unstructured play during the school day. When children are given 60 min of recess per day, they will meet the daily recommended 60 min of MVPA during school alone. Recess could be a vital piece in reducing sedentary behavior and obesity rates seen in children across the country today. These findings support the LiiNK intervention as an effective method to improve PA patterns in elementary school children. Schools across the country should try to add additional time for recess during the school day instead of taking it away from their students.

## Figures and Tables

**Table 1:** Descriptive Statistics. Means and standard deviations of moderate and vigorous physical activity (MVPA) and steps for grade, gender, and total.

Descriptive Statistics										
	Steps		Minutes in Sedentary		Minutes in Light		Minutes in Moderate		Minutes in Vigorous	
	Mean	SD	Mean	SD	Mean	SD	Mean	SD	Mean	SD
Grade										
1st Grade	8181.96	964.40	124.50	26.81	164.30	14.47	103.95	18.88	27.25	9.84
2nd Grade	8644.84	1080.71	122.91	24.13	159.47	13.74	108.62	18.32	29.00	11.04
Gender										
Male	8628.09	1093.38	123.52	24.11	161.55	14.20	105.17	17.40	29.76	11.25
Female	8202.83	961.32	123.92	26.82	162.33	14.28	107.17	19.87	26.58	9.47
Total	8406.03	1045.14	123.73	25.48	161.96	14.28	106.21	18.70	28.10	10.44
